# *N*-[4-(Prop-2-yn­yloxy)phen­yl]maleimide

**DOI:** 10.1107/S1600536808035885

**Published:** 2008-12-10

**Authors:** Zhan-Xian Li, Cai-Mei Ren, Sen Yang, Guang-Jun Yao, Qiu-Zhi Shi

**Affiliations:** a Department of Chemistry, Zhengzhou University, Zhengzhou 450001, People’s Republic of China

## Abstract

In the title compound, C_13_H_9_NO_3_, the dihedral angle between the benzene and maleimide rings is 64.1 (2)°. In the crystal structure, mol­ecules interact *via* C—H⋯O inter­actions.

## Related literature

*N*-substituted maleimides can be used in free-radical-initiated polymerization processes upon exposure to light, see: Chang *et al.* (1999 ▶); Hoyle *et al.* (1999 ▶). For related structures, see: Moreno-Fuquen *et al.* (2006 ▶, 2008*a* ▶,*b* ▶). For the effect of benzene ring substituents on the dihedral angle between the benzene and imidic rings, see: Miller *et al.* (2000 ▶).
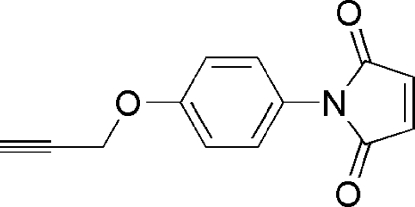


## Experimental

### 

#### Crystal data

C_13_H_9_NO_3_
*M* *_r_* = 227.21
Monoclinic, 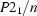

*a* = 9.0428 (18) Å
*b* = 11.491 (2) Å
*c* = 11.492 (2) Å
β = 102.00 (3)°
*V* = 1168.0 (4) Å^3^
*Z* = 4
Mo *K*α radiation
μ = 0.09 mm^−1^
*T* = 292 (3) K
0.30 × 0.26 × 0.20 mm

#### Data collection

Bruker SMART 1K CCD area-detector diffractometer
Absorption correction: multi-scan (*SADABS*; Bruker, 2000 ▶) *T*_min_ = 0.968, *T*_max_ = 0.981
6107 measured reflections
2053 independent reflections
1368 reflections with *I* > 2σ(*I*)
*R*_int_ = 0.088

#### Refinement

*R*[*F* ^2^ > 2σ(*F* ^2^)] = 0.072
*wR*(*F* ^2^) = 0.224
*S* = 1.35
2053 reflections
154 parameters
H-atom parameters constrained
Δρ_max_ = 0.48 e Å^−3^
Δρ_min_ = −0.43 e Å^−3^

### 

Data collection: *SMART* (Bruker, 2000 ▶); cell refinement: *SAINT* (Bruker, 2000 ▶); data reduction: *SAINT*; program(s) used to solve structure: *SHELXTL* (Sheldrick, 2008 ▶); program(s) used to refine structure: *SHELXTL*; molecular graphics: *SHELXTL*; software used to prepare material for publication: *SHELXTL*.

## Supplementary Material

Crystal structure: contains datablocks I, global. DOI: 10.1107/S1600536808035885/hg2436sup1.cif

Structure factors: contains datablocks I. DOI: 10.1107/S1600536808035885/hg2436Isup2.hkl

Additional supplementary materials:  crystallographic information; 3D view; checkCIF report

## Figures and Tables

**Table 1:** Hydrogen-bond geometry (Å, °)

*D*—H⋯*A*	*D*—H	H⋯*A*	*D*⋯*A*	*D*—H⋯*A*
C2—H2⋯O2^i^	0.93	2.50	3.179 (12)	130
C9—H9⋯O2^ii^	0.93	2.18	3.103 (10)	169

Symmetry codes: (i) 
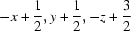
; (ii) 
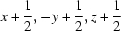
.

**Fig. 1.**
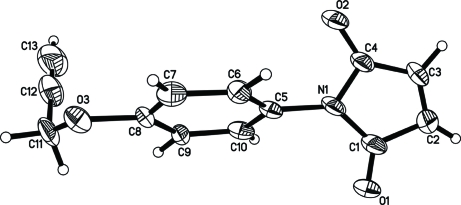
A view of complex (I), showing 30% probability displacement ellipsoids and the atom-numbering scheme.

## supplementary crystallographic information

### Comment

N-substituted maleimides can be used in free radical initiated polymerization process upon exposure to light (Chang, *et al.*, 1999; Hoyle, *et al.*,1999). *N*-(3-Nitrophenyl)maleimide (Moreno-Fuquen, *et al.*, 2006), *N*-(3-Chlorophenyl)maleimide (Moreno-Fuquen, *et al.*, 2008a) and *N*-(4-Chlorophenyl)maleimide (Moreno-Fuquen, *et al.*, 2008b) has reported and could be taken as a reference to compare with the structural characteristics of (I).

Perspective view of (I), showing the atomic numbering scheme, can be seen in Fig. 1. The dihedral angle between the benzene and imidic rings influences on the polymerization process, and subsituents of the benzene ring can effect the value of dihedral angle (Miller *et al.* 2000). In the title compound (I), the dihedral angle between the benzene and maleimide is 64.1 (2)°. This angle is 46.46 (5) ° for *N*-(3-Chlorophenyl)maleimide (Moreno-Fuquen, *et al.*, 2008:1) and 47.54 (9) ° for *N*-(4-Chlorophenyl)maleimide (Moreno-Fuquen, *et al.*, 2008:2). The crystal structure of (I) is stabilized by weak intermolecular C—H···O hydrogen bonds.

### Experimental

The title compound was prepared by taking equimolar quantities of 4-(prop-2-ynyloxy)benzenamine (14.7 g, 0.1 mol) and maleic anhydride (9.8 g, 0.1 mol) in 80 ml benzene and 20 ml DMF and refluxing 4 h in the presence of *p*-toluenesulfonic acid. The reaction product was poured into 500 ml ice water, yellow precipitate product was formed. The crude product was recrystalled from ethanol. Yield 90%. ^1^H NMR (300 MHz, DMSO-d_6_) δ 7.30, 7.13(d, aromatic), 7.02(s, 2H, maleimide), 4.85(s, 2H, –H_2_–), 3.14(s, 1H, ≡-H). Analysis. calculated for C_13_H_9_NO_3_: C 68.72, H 3.99, N 6.16%. Found: C 68.39, H 4.02, N 6.21%. The product added in 50 ml ethanol and crystals of (I) suitable for X-ray analysis were obtained by slow evaporation at room temperature.

### Refinement

The H atoms bound to C atoms were placed in caculated positions with C—H = 0.93 Å and included in the refinement with *U*_iso_(H) = 1.2*U*_eq_(C).

### Crystal data

**Table:**

C_13_H_9_NO_3_	*F*(000) = 472
*M**_r_* = 227.21	*D*_x_ = 1.292 Mg m^−^^3^
Monoclinic, *P*2_1_/*n*	Mo *K*α radiation, λ = 0.71073 Å
Hall symbol: -P 2yn	Cell parameters from 2053 reflections
*a* = 9.0428 (18) Å	θ = 2.5–25.0°
*b* = 11.491 (2) Å	µ = 0.09 mm^−^^1^
*c* = 11.492 (2) Å	*T* = 292 K
β = 102.00 (3)°	Block, yellow
*V* = 1168.0 (4) Å^3^	0.30 × 0.26 × 0.20 mm
*Z* = 4	

### Data collection

**Table:**

Bruker SMART 1K CCD area-detector diffractometer	2053 independent reflections
Radiation source: fine-focus sealed tube	1368 reflections with *I* > 2σ(*I*)
graphite	*R*_int_ = 0.088
phi and ω scans	θ_max_ = 25.0°, θ_min_ = 2.5°
Absorption correction: multi-scan (*SADABS*; Bruker, 2000)	*h* = −10→10
*T*_min_ = 0.968, *T*_max_ = 0.981	*k* = −13→10
6107 measured reflections	*l* = −12→12

### Refinement

**Table:**

Refinement on *F*^2^	Primary atom site location: structure-invariant direct methods
Least-squares matrix: full	Secondary atom site location: difference Fourier map
*R*[*F*^2^ > 2σ(*F*^2^)] = 0.072	Hydrogen site location: inferred from neighbouring sites
*wR*(*F*^2^) = 0.224	H-atom parameters constrained
*S* = 1.35	*w* = 1/[σ^2^(*F*_o_^2^) + (0.0733*P*)^2^ + 3.1182*P*] where *P* = (*F*_o_^2^ + 2*F*_c_^2^)/3
2053 reflections	(Δ/σ)_max_ < 0.001
154 parameters	Δρ_max_ = 0.49 e Å^−^^3^
0 restraints	Δρ_min_ = −0.43 e Å^−^^3^

### Special details

**Table:**

Geometry. All e.s.d.'s (except the e.s.d. in the dihedral angle between two l.s. planes) are estimated using the full covariance matrix. The cell e.s.d.'s are taken into account individually in the estimation of e.s.d.'s in distances, angles and torsion angles; correlations between e.s.d.'s in cell parameters are only used when they are defined by crystal symmetry. An approximate (isotropic) treatment of cell e.s.d.'s is used for estimating e.s.d.'s involving l.s. planes.
Refinement. Refinement of *F*^2^ against ALL reflections. The weighted *R*-factor *wR* and goodness of fit *S* are based on *F*^2^, conventional *R*-factors *R* are based on *F*, with *F* set to zero for negative *F*^2^. The threshold expression of *F*^2^ > σ(*F*^2^) is used only for calculating *R*-factors(gt) *etc*. and is not relevant to the choice of reflections for refinement. *R*-factors based on *F*^2^ are statistically about twice as large as those based on *F*, and *R*- factors based on ALL data will be even larger.

### Fractional atomic coordinates and isotropic or equivalent isotropic displacement parameters (Å^2^)

**Table:**

	*x*	*y*	*z*	*U*_iso_*/*U*_eq_	
C3	0.2361 (11)	0.5326 (8)	0.6724 (8)	0.088 (4)	
H3	0.2039	0.5394	0.5904	0.106*	
O3	0.0885 (6)	0.1202 (5)	1.2278 (5)	0.0694 (19)	
C5	0.1785 (7)	0.3652 (6)	0.9539 (6)	0.052 (2)	
O1	0.3695 (7)	0.5806 (5)	0.9313 (5)	0.083 (2)	
O2	0.0834 (8)	0.3616 (6)	0.7229 (6)	0.094 (3)	
C8	0.1352 (8)	0.1986 (7)	1.1335 (6)	0.048 (2)	
C9	0.2725 (8)	0.2328 (7)	1.0900 (6)	0.054 (2)	
H9	0.3586	0.1960	1.1323	0.065*	
C10	0.3076 (7)	0.3112 (7)	0.9958 (6)	0.053 (2)	
H10	0.3992	0.3200	0.9720	0.064*	
C7	0.0089 (9)	0.2487 (8)	1.0914 (8)	0.066 (3)	
H7	−0.0834	0.2363	1.1129	0.079*	
C6	0.0429 (7)	0.3326 (7)	0.9989 (7)	0.059 (2)	
H6	−0.0424	0.3736	0.9613	0.071*	
C1	0.3150 (9)	0.5431 (8)	0.8502 (7)	0.062 (3)	
C4	0.1745 (10)	0.4410 (7)	0.7421 (7)	0.070 (3)	
C11	0.2080 (11)	0.0481 (9)	1.2619 (8)	0.077 (3)	
H11A	0.1862	0.0016	1.3268	0.093*	
H11B	0.2935	0.0971	1.2958	0.093*	
C2	0.3280 (11)	0.5958 (8)	0.7276 (8)	0.078 (3)	
H2	0.3866	0.6553	0.7059	0.094*	
C12	0.2667 (12)	−0.0409 (10)	1.1735 (10)	0.081 (3)	
C13	0.3170 (15)	−0.1138 (12)	1.1030 (12)	0.113 (5)	
H13	0.3526	−0.1656	1.0530	0.136*	
N1	0.2168 (7)	0.4475 (6)	0.8557 (5)	0.0543 (19)	

### Atomic displacement parameters (Å^2^)

**Table:**

	*U*^11^	*U*^22^	*U*^33^	*U*^12^	*U*^13^	*U*^23^
C3	0.115 (8)	0.070 (6)	0.055 (6)	0.005 (6)	−0.038 (6)	0.016 (5)
O3	0.068 (4)	0.078 (4)	0.052 (3)	0.000 (3)	−0.010 (3)	0.011 (3)
C5	0.053 (4)	0.053 (5)	0.038 (4)	−0.006 (4)	−0.017 (4)	−0.001 (4)
O1	0.093 (5)	0.077 (4)	0.058 (4)	−0.021 (4)	−0.038 (4)	−0.001 (3)
O2	0.107 (5)	0.068 (4)	0.074 (4)	−0.006 (4)	−0.056 (4)	0.003 (3)
C8	0.064 (5)	0.049 (5)	0.026 (4)	−0.009 (4)	−0.003 (4)	0.009 (3)
C9	0.059 (5)	0.054 (5)	0.039 (4)	−0.021 (4)	−0.010 (4)	0.016 (4)
C10	0.039 (4)	0.068 (5)	0.045 (5)	−0.006 (4)	−0.007 (4)	−0.009 (4)
C7	0.045 (5)	0.075 (6)	0.069 (6)	−0.013 (4)	−0.006 (4)	0.001 (5)
C6	0.057 (5)	0.051 (5)	0.058 (5)	−0.002 (4)	−0.014 (4)	0.007 (4)
C1	0.057 (5)	0.069 (6)	0.047 (5)	0.002 (4)	−0.018 (4)	−0.003 (5)
C4	0.082 (6)	0.052 (5)	0.054 (5)	0.013 (5)	−0.034 (5)	−0.002 (4)
C11	0.082 (6)	0.096 (8)	0.042 (5)	−0.010 (6)	−0.012 (5)	0.036 (5)
C2	0.088 (7)	0.058 (6)	0.072 (6)	−0.011 (5)	−0.020 (5)	0.016 (5)
C12	0.085 (7)	0.083 (8)	0.068 (7)	0.019 (6)	0.000 (6)	0.028 (6)
C13	0.134 (11)	0.098 (9)	0.092 (9)	0.023 (9)	−0.014 (8)	0.019 (8)
N1	0.051 (4)	0.064 (4)	0.036 (4)	0.004 (3)	−0.018 (3)	0.000 (3)

### Geometric parameters (Å, °)

**Table:**

C3—C2	1.184 (11)	C10—H10	0.9300
C3—C4	1.498 (9)	C7—C6	1.513 (11)
C3—H3	0.9300	C7—H7	0.9300
O3—C11	1.353 (10)	C6—H6	0.9300
O3—C8	1.535 (9)	C1—N1	1.422 (11)
C5—C10	1.321 (7)	C1—C2	1.560 (12)
C5—C6	1.475 (8)	C4—N1	1.284 (10)
C5—N1	1.565 (10)	C11—C12	1.608 (16)
O1—C1	1.052 (9)	C11—H11A	0.9700
O2—C4	1.218 (10)	C11—H11B	0.9700
C8—C7	1.281 (10)	C2—H2	0.9300
C8—C9	1.484 (8)	C12—C13	1.311 (16)
C9—C10	1.493 (10)	C13—H13	0.9300
C9—H9	0.9300		
			
C2—C3—C4	116.3 (8)	C7—C6—H6	112.3
C2—C3—H3	121.9	O1—C1—N1	117.3 (9)
C4—C3—H3	121.9	O1—C1—C2	122.1 (9)
C11—O3—C8	104.1 (6)	N1—C1—C2	120.4 (7)
C10—C5—C6	119.3 (7)	O2—C4—N1	106.0 (8)
C10—C5—N1	103.6 (6)	O2—C4—C3	137.9 (8)
C6—C5—N1	136.9 (6)	N1—C4—C3	115.9 (8)
C7—C8—C9	119.8 (7)	O3—C11—C12	123.7 (7)
C7—C8—O3	100.1 (7)	O3—C11—H11A	106.4
C9—C8—O3	139.9 (6)	C12—C11—H11A	106.4
C8—C9—C10	136.3 (6)	O3—C11—H11B	106.4
C8—C9—H9	111.9	C12—C11—H11B	106.4
C10—C9—H9	111.9	H11A—C11—H11B	106.5
C5—C10—C9	104.1 (6)	C3—C2—C1	93.9 (8)
C5—C10—H10	128.0	C3—C2—H2	133.0
C9—C10—H10	128.0	C1—C2—H2	133.0
C8—C7—C6	104.9 (7)	C13—C12—C11	178.9 (10)
C8—C7—H7	127.6	C12—C13—H13	180.0
C6—C7—H7	127.6	C4—N1—C1	93.2 (7)
C5—C6—C7	135.5 (6)	C4—N1—C5	129.4 (7)
C5—C6—H6	112.3	C1—N1—C5	137.1 (5)
			
C11—O3—C8—C7	169.0 (7)	C4—C3—C2—C1	−4.4 (12)
C11—O3—C8—C9	−16.0 (12)	O1—C1—C2—C3	−172.0 (11)
C7—C8—C9—C10	−5.8 (14)	N1—C1—C2—C3	2.3 (12)
O3—C8—C9—C10	179.9 (8)	O2—C4—N1—C1	−179.3 (7)
C6—C5—C10—C9	−4.1 (9)	C3—C4—N1—C1	−3.7 (9)
N1—C5—C10—C9	179.7 (5)	O2—C4—N1—C5	6.4 (12)
C8—C9—C10—C5	6.6 (12)	C3—C4—N1—C5	−178.0 (7)
C9—C8—C7—C6	1.8 (10)	O1—C1—N1—C4	175.7 (9)
O3—C8—C7—C6	178.1 (6)	C2—C1—N1—C4	1.2 (10)
C10—C5—C6—C7	2.5 (14)	O1—C1—N1—C5	−10.7 (15)
N1—C5—C6—C7	177.1 (8)	C2—C1—N1—C5	174.7 (8)
C8—C7—C6—C5	−0.8 (13)	C10—C5—N1—C4	109.2 (9)
C2—C3—C4—O2	−179.8 (12)	C6—C5—N1—C4	−65.9 (13)
C2—C3—C4—N1	6.5 (14)	C10—C5—N1—C1	−62.5 (11)
C8—O3—C11—C12	−61.5 (10)	C6—C5—N1—C1	122.4 (10)

### Hydrogen-bond geometry (Å, °)

**Table:**

*D*—H···*A*	*D*—H	H···*A*	*D*···*A*	*D*—H···*A*
C2—H2···O2^i^	0.93	2.50	3.179 (12)	130
C9—H9···O2^ii^	0.93	2.18	3.103 (10)	169

Symmetry codes: (i) −*x*+1/2, *y*+1/2, −*z*+3/2; (ii) *x*+1/2, −*y*+1/2, *z*+1/2.
